# Supplementary material for: Programmable synthetic cell networks regulated by tuneable reaction rates
Source: Nat Commun. 2022 Jul 6;13:3885. doi: 10.1038/s41467-022-31471-5 (PMC9259615; doi:10.1038/s41467-022-31471-5)
Supplement: Supplementary file 1 — Supplementary information [file 41467_2022_31471_MOESM1_ESM.pdf]

Supplementary information: Programmable synthetic cell networks regulated by tuneable reaction rates

T-Y D Tang et al.

Supplementary Methods

Materials

Synperonic ® F 108 surfactant, DL-Dithiothreitol (DTT, MW = 154.2 g.mol^{-1}, ≥98% TLC and ≥99% titration), Netropsin dihydrochloride from *Streptomyces netropsis* powder (MW = 503.4 g.mol^{-1}, ≥98%, HPLC and TLC), Glucose oxidase from *Aspergillus niger* (GOx, MW = 160 kDa), Bovine serum albumin lyophilized powder (BSA, MW = 66 kDa ≥96%), alcohol dehydrogenase from *Saccharomyces cerevisiae* (ADH, MW = 141 kDa), formate dehydrogenase from *Candida boidinii* (FDH, MW (monomer) = 41 kDa, and 2-ethyl-1-hexanol (≥98%), anhydrous dimethyl sulfoxide (MW = 78.1 g.mol^{-1}, ≥99.9%), N-isopropylacrylamide (NIPAAm, MW = 113.2 g.mol^{-1}, 98 %) and n-hexane (MW = 86.2 g.mol^{-1}, reagent grade, ≥99%), monopotassium phosphate (KH_2PO_4) (MW = 136.1 g.mol^{-1}), Tris-HCl (MW = 121.4 g.mol^{-1}), sodium hydrogen carbonate ($NaHCO_3$, MW = 84 g.mol^{-1}), chlorotrimethylsilane (MW = 108.6 g.mol^{-1}) and Hellmanex® III were purchased from Sigma-Aldrich (Missouri, USA) and used without further purification. Ammonium sulphate ($(NH_4)_2SO_4$, MW = 132 g.mol^{-1}), potassium chloride (KCl, MW = 74.6 g.mol^{-1}), magnesium sulphate ($MgSO_4$, MW = 120.4 g.mol^{-1}), sodium chloride (NaCl, MW = 58.4g.mol^{-1}), Trizma hydrochloride (Tris-HCl, MW = 157.6 g.mol^{-1}), hydrochloric acid (34.46 g.mol^{-1}) and Amicon ultrafilters (10 kDa and 30 kDa cut-off), unconjugated streptavidin from *Streptomyces avidinii (*MW ~ 60 kDa*)* were all purchased from Merck (Darmstadt, Germany). Amine-reactive fluorescein 5-isothiocyanates isomer I (FITC, MW = 389 Da) and Zeba^{TM} spin columns (MW cut-off 7 kDa and 40 kDa) and Pierce™ BCA protein assay kit were purchased from Thermofisher Scientific (Massachusetts, USA). Methanol and N,N-Dimethylacetamide was purchased from VWR chemicals (Pensylvannia, USA). Toluene (MW = 92.14 g.mol^{-1}) was purchased from Carl Roth GmbH (Karlsruhe, Germany). 3-[Methoxy(polyethyleneoxy) propyl]trimethoxysilane (MW = 1120-1250 g.mol^{-1}) was purchased from abcr GmbH (Karlsruhe, Germany). LiCl was purchased from Honeywell (North Carolina, USA), Poly(2-vinylpyridin) was purchased from Polymer Standards Service (Mainz, Germany). EvaGreen® dye (in water) was obtained from Biotium (Freemont, USA). DNA strands (unmodified, with phosphorothioate modifications or tagged with biotin-TEG or DY530) were purchased from Biomers GmbH (Ulm, Germany) with HPLC purification. DNA and RNA strands labelled with FAM on the 5' end (CATTCTGACGAG, TCGAGTCTGTT, GCACUUCGGUGC) were purchased from Eurofins Scientific (Luxembourg). Bst DNA polymerase large fragment (67 kDA, 8000 units/ml, 7.76 µg/ml), Nb.Bsml nickase (~78 kDa, 20.9 µg/ml, 10,000 units.mL^{-1}) and deoxynucleotide solution mix (dNTP, 10 mM each) were purchased from New England Biolabs (NEB, Massacussets, USA). The polymerase was diluted 5X in storage buffer (Supplementary Table 5) and stored at -20 °C until further use. The recombinant *Thermus thermophilus* ttRecJ exonuclease (73 kDa) was a kind gift from Dr. André Estevez-Torres (CNRS and Sorbonne Université, Paris, France) and produced as described[1]. Monomeric Green fluorescent protein (monoGFP, MW = 25 kDa), were a kind gift from the Protein Expression Purification and Characterisation facility (PEPC), MPI-CBG, Dresden, Germant. PEGylated bis(sulfosuccinimidyl)suberate ($BS(PEG)_9$, MW = 708 g.mol^{-1}) purchased from ThermoFisher Scientific was dissolved in anhydrous dimethyl sulfoxide, aliquoted and stored under argon gas at -20°C until use. EPOXY resin and hardener (1:1 ratio), was purchased from R&G Faserverbundwerkstoffe (Waldenbuch, Germany) and used as directed by the manufacturer's instructions. F-Boden Black 96 well and Flat Black 384

well plates were purchased from Greiner Bio-One, (Kremuenster, Austria). Hollow rectangle capillaries were purchased from Vitrocom (New Jersey, USA). Silicon wafer was purchased by Silicon Materials, Germany. SU-8 2010 photoresist was purchased from Microchem (Texas, USA) and polydimethylsiloxane was purchased from Dow Corning, (Michigan, USA). Capillary, parafilm based custom made glass slides were prepared as described below with glass slides (Thermofisher Scientific, Massachusetts, USA, 26 x 76 mm), precision coverglass (Paul Marienfeld GmbH Co. KG, Lauda-Königshofen, Germany, 22 x 22 mm, No. 1.5H), parafilm (Bemis, Wisconsin, USA), and Twinsil quick (Dental-Produktions- und Vertriebs-GmbH, Wipperfürth, Germany). Borosilicate glass hollow rectangular capillaries (inner diameter: 0.1 mm, length 1 mm) were purchased from CM scientific, Republic of Ireland.

oligo	sequence	5' modification	3' modification	Type
T_2	C*T*C*G*TCAGAATGCTCGTCAGAATG	Biotin-TEG	Dy530	Autocatalytic template
S_2	CATTCTGACGAG	None	None	Substrate for autocatalytic reaction
S_1	CATTCAGGATCG			Substrate for linear reaction
T_1	CGATCCTGAATG-CTCGTCAGAATG	Biotin-TEG	ROX	Linear activation template
	CATTCTGACGAG	FAM	None	Substrate for partitioning experiments
	TCGAGTCTGTT	FAM	None	Substrate for partitioning experiments
DNA for degradation	CTCGTCAGAATGCTCGTCAGAATG		Biotin-TEG	Template for degradation
DNA FRAP	CTCGTCAGAATGCTCGTCAGAATG	Biotin-TEG	DY530	

Supplementary Table 1. The DNA sequences used in this work. * denotes phosphothiorionate modifications which prevents exonuclease degradation.

Preparation of Silanated glass sample chambers:

Silanated coverslips were prepared by washing glass coverslips (22 x 22 mm, No. 1.5H) with Hellmanex® III according to the manufacturor's instructions and then incubated with 4.6 $mg.L^{-1}$ 3-

[Methoxy(polyethyleneoxy)propyl]trimethoxysilane in toluene (supplemented with 0.08% v/v of 37% HCl) for 18 hrs at room temperature. After incubation, the coverslips were washed first with toluene, then twice each with ethanol and ultrapure water. Finally, the coverslips were dried with compressed air. The coverslips were then assembled onto glass slides with parafilm. In brief, parafilm, cut into 2 x 26 mm stripes, was placed onto an ethonal-cleaned 26x76 mm coverslide to produce channels. The silanated cover slip was pressed onto the precut parafilm layer and then heated on a hot plate set to 90 °C to melt the parafilm and adhere the coverslip to the glass slide. The custom made channels slides were left to cool at room temperature.

Determination of the degree of molecular sequestration

The degree of sequestration of Fluorescein Isothiocyanate (FITC) -labelled Alcohol dehydrogenase (ADH), Bovine Serum Albumin (BSA), Formate dehydrogenase (FDH), ttRecJ, GFP, FAM-labelled DNA (CATTCTGACGAG, TCGAGTCTGTT) was estimated by determination of the fluorescence intensity of FITC inside and outside of the droplet with background removal from proteinosome only with the DNA/ streptavidin constructs.

$$K = \frac{F_{in}}{F_{out}} \qquad (3)$$

Where F_{in} is the fluorescence intensity inside the proteinosomes and F_{out} is the fluorescence intensity outside of the proteinosome.

To do this, proteins were labelled with FITC by firstly, exchanging the buffer of the proteins into into freshly prepared $NaHCO_3$ buffer (0.2 M, pH 8.4) and then mixed with a 50x molar excess of FITC. The solution was incubated in the dark for 2 hrs with rotation to ensure efficient mixing. The unbound dyes were then removed from the solution by exchanging the buffer with Trizma® Hydrochloride (0.1 M, pH 8.0). Buffer exchange steps were performed with Zeba™ spin columns (7 kDa cut off for BSA and FDH or 40 kDa cut off for all other proteins). The final concentration of the protein and the protein to dye ratio was determined by UV-vis absorbance using the Nanodrop 1000 (Thermofisher scientific, Massachusetts USA) at 280 nm and 494 nm.

To determine the sequestration of fluorescently labelled molecules into proteinosomes. Proteinsomes containing ROX-labelled T_1 DNA-biotin-streptavidin complexes or Alexa Fluor 594 (AF594)- labelled T_2 DNA-biotin streptavidin complexes were diluted into a reaction solution without EvaGreen or PEN enzymes. AF594 T_2 DNA-biotin streptavidin complexes were used in lieu of DY530 labelled DNA due to the overlap in fluorescence excitation and emission with FITC labelled substrates. Fluorescent molecules were added to the dispersion of proteinosomes to following final total concentrations (ADH (0.4 µM, 0.06 mg.mL^{-1}), BSA (2.58 - 38.64 µM, 0.17 – 2.55 mg.mL^{-1}), FDH (4.55 µM , 0.2 mg.mL^{-1}), ttRecJ (1.23 µM, 0.09 mg.mL^{-1}), and monoGFP (2 - 20 µM, 0.05 - 0.5 mg.mL^{-1}) and FAM labelled DNA (CATTCTGACGAG, TCGAGTCTGTT, 10 µM) to achieve a final reaction solution of 1x. After 5 mins of incubation, the samples were loaded in homemade silanated customised parafilm channels prepared on glass slides. Both ends of the channel were sealed with Twinsil® quick two-component silicon glue and incubated at room temperature to allow curing of the glue and then at 42˚C for 10 minutes on the microscope stage before imaging.

The samples were imaged using a Zeiss LSM 880 inverted single point scanning confocal microscope equipped with a 32 GaAsP PMT channel spectral detector and imaged using a 20x objective (Plan-Apochromat 20x/ 0.8 objective, Zeiss) at 42°C. A tile scan of 3 by 5 or 4 by 4 images (354.25 x 354.25 μm) was obtained with 10% overlap with $\lambda^{exc}_{ROX/AF594}$= 561 nm $\lambda^{emi}_{ROX/AF594}$ = 590-650 nm, $\lambda^{exc}_{FITC\ /\ FAM\ /GFP}$= 488 nm $\lambda^{emi}_{FITC\ /\ FAM\ /GFP}$ = 490-560 nm.

Optical microscopy images were analysed using Fiji[2]. A Fiji script was used to detect proteinosomes based on the fluorescence from the AF594 or Rox-labelled DNA (see Fiji code). Extracted ROIs were used to extract signals from both AF594/ROX and FITC/FAM/GFP channel. Partitioning coefficient was calculated by fluorescent signal inside proteinosome divided by fluorescent signal in the buffer with background subtraction using proteinosomes containing ROX-labelled T_1 or AF594 labelled T_2 DNA-biotin-streptavidin complexes. The partition coefficient was determined for at least 30 different proteinosomes and the average and standard deviation obtained.

Determination of DNA concentration within proteinosomes after microfluidic encapsulation.

To determine the encapsulation efficiency of DNA-biotin-streptavidin complex within proteinosomes prepared by microfluidics a calibration curve for DY530- labelled DNA- biotin-streptavidin was undertaken using the Zeiss LSM 880 single point scanning confocal microscope using a 40x objective (C-Apochromat 40x NA 1.2W objective, Zeiss). To do this, a serial dilution of DY530-labelled DNA-biotin-streptavidin at concentrations of 0, 0.031, 0.063, 0.13, 0.25, 0.5 μM DNA were prepared in water and loaded into custom made channel slides. The samples were loaded onto the microscope and incubated at 25˚ C for 30 mins to ensure temperature equilibration. The microscope settings were consistent with those used for the autocatalytic and linear kinetic experiments (Figure 3) (λ^{exc}_{DY530}= 514 nm, λ^{emi}_{DY530} = 543-695 nm) and for the proteinosomes prepared for the effect of encapsulation on the autocatalytic reaction respectively.

The images were analysed using a FIJI macro code and the average fluorescence intensity at known DY530-DNA-biotin-streptavidin concentration was obtained and plotted. The data was fit to a linear regression equation to obtain the following equation Fluorescence (RFU) = 3940.7 x (DNA concentration in μM) + 58.4 (R^2 = 0.9986) and used to convert fluorescence intensity within proteinosomes into concentrations. A Gaussian fit was applied to the histogram of number of proteinosomes vs concentration to obtain the average concentration and a standard deviation from 94 proteinosomes. 0.12 μM with a std=0.017 μM).

Determination of protein concentration and PNIPAAm labelling ratio

The PNIPAAm-conjugated protein ratio used for the partitioning assay was quantified using the Pierce BCA protein assay kit according to the manufactor's instruction. The corresponding PNIPAAm concentration was measured via absorbance at 300 nm via a calibration curve with PNIPAAm dilutions. Both measurements were prepeared in 96-well plates and measured on Spark 20 M well plate spectrophotometer (Tecan AG, Männedorf, Switzerland).

Buffers

Buffer A at 10 x, pH 8.8	Concentration in 10x
Tris-HCl (pH 9)	200 mM
$(NH_4)_2SO_4$	100 mM
KCl	100 mM
$MgSO_4$	20 mM

Supplementary Table 2. Buffer A was prepared typically at 10 times the final concentration containing the salts listed in the table above.

Reaction buffer at 1x, pH 8.6	Final concentration in 1 x
Buffer A	1 x
NaCl	50 mM
$MgSO_4$	6 mM
Synperonic F108	1 $g.L^{-1}$
Netropsin	1 µM

Supplementary Table 3. The reaction buffer was prepared typically at 7 times the final concentration. However, the table above lists the component of this buffer at their final concentrations (1x)

Reaction solution	Concentration in final reaction solution
Reaction buffer	1x
DL-Dithiothreitol (DTT)	3 mM
BSA native	0.5 $mg.mL^{-1}$
EvaGreen dye (20 x)	2.2 x
dNTP mix solution	0.1 mM
DNA template or<br>proteinosomes with 0.12 µM DNA template	0 – 1000 nM<br>0 – 20 % (v/v)
S2	1 nM
Nb.BsmI nickase	400 $units.mL^{-1}$
Bst DNA polymerase	12.8 $units.mL^{-1}$
ttRecJ exonuclease	0.6 nM

Supplementary Table 4. A typical reaction solution.

Salt	Concentration ($mmol.L^{-1}$)
NaCl	137
KCl	2.7
Na_2HPO_4	10
KH_2PO_4	1.8

Supplementary Table 5. 1x Phosphate-buffered saline (PBS) buffer was prepared at a pH 7.4 as described in the table above.

Storage buffer for Bst DNA polymerase	Concentration in final reaction solution
Trizma base (pH 7.4 with HCl)	10 mM
KCl	50 mM
DTT	1 mM
EDTA	0.1 mM
Triton®X-100	0.1%
Glycerol	50 %
pH adjusted to 7.1 at 25˚C	

Supplementary Table 6. Storage buffer for Bst DNA polymerase

Buffer for proteinosome	1x solution
HEPES	50 mM
$MgCl_2$	10 mM
KCl	100 mM
KOH	Used to adjust to pH 7.6

Supplementary Table 7. Buffer for Proteinosome preparation

FIJI code

```
// Segmentation of Droplets
selectWindow("name");
run("Duplicate...", " ");
name=getTitle;
run("Gaussian Blur...", "sigma=1.30");
run("Threshold...");
setAutoThreshold("Default dark");
run("Convert to Mask");
run("Watershed");
run("Analyze Particles...", "size=250-9160 circularity=0.7 exclude add in_situ");

// Isolating proteinosomes
run("Clear Results");
roiManager("reset");
run("Duplicate...");
run("Gaussian Blur...", "sigma=2");
run("8-bit");
run("Threshold...");
waitForUser("set the threshold and press OK, or cancel to exit macro");
run("Convert to Mask");
run("Watershed");
run("Analyze Particles...", "size=200-3000 circularity=0.70-1.00 exclude include add in_situ");
//size is pre-measured  with 100 -  Inf in psome_in_water group
```

Supplementary figures

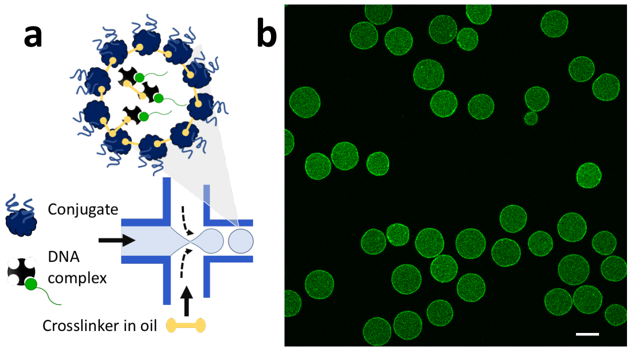

Supplementary Figure 1. High throughput production of proteinosomes (a) A schematic illustration of the flow-focusing device used for the formation of proteinosomes. Water-in-oil emulsions are produced at the flow focus junction by flowing an aqueous solution of protein conjugate (4mg.mL^{-1}) and DNA complex (1 μM) with 2-ethyl-1-hexanol containing the crosslinker BS(PEG)$_9$ (0.5–2 mM). (b) Confocal imaging of proteinosomes containing DNA complex labelled with DY530. Scale bar is 20 μm.

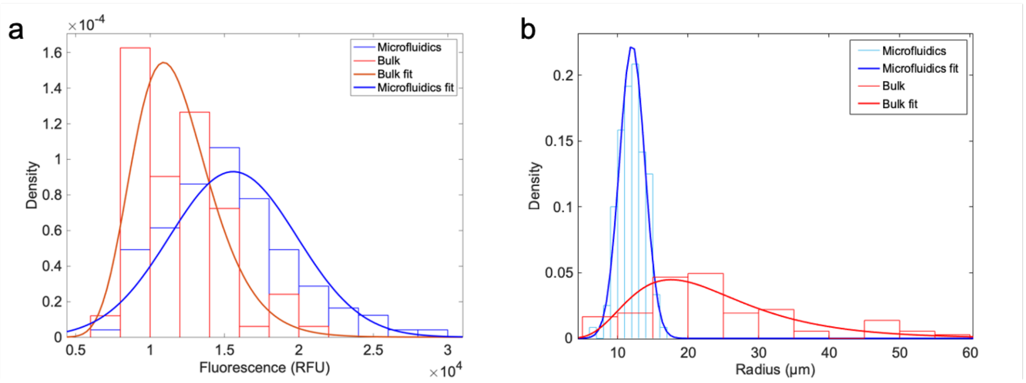

Supplementary Figure 2. Distribution of size (a) and DNA concentration (b) for bulk-assembled (red) and microfluidic-assembled proteinosomes (blue) (a) The fluorescence probability density showed a log-normal and a normal distribution (respectively) for proteinosomes produced by bulk methodologies (F_{mean} = 1.2 x 10^4 ± 2.7 x 10^3) (red) and microfluidic (F_{mean} = 1.5 x 10^4 ± 4.6 x 10^3) (blue). (b) The radius probability density showed a normal distribution for proteinosomes produced by bulk (r_{mean} = 24.0 ± 11.4 µm) (red) and microfluidic (r_{mean} = 12.1 ± 1.8 µm) (blue) methodologies. Confocal microscopy images of proteinosomes (in 1 x HEPES buffer, pH 7.6 containing DY-530 tagged DNA were analysed using FIJI-imageJ scripts. Images were acquired using a Zeiss LSM 700 inverted confocal microscope using a 40x (1.2 W) objective at room temperature. Bold lines are Gaussian fits which are used as a guide and not for fitting purposes. Source data are provided as a Source data file.

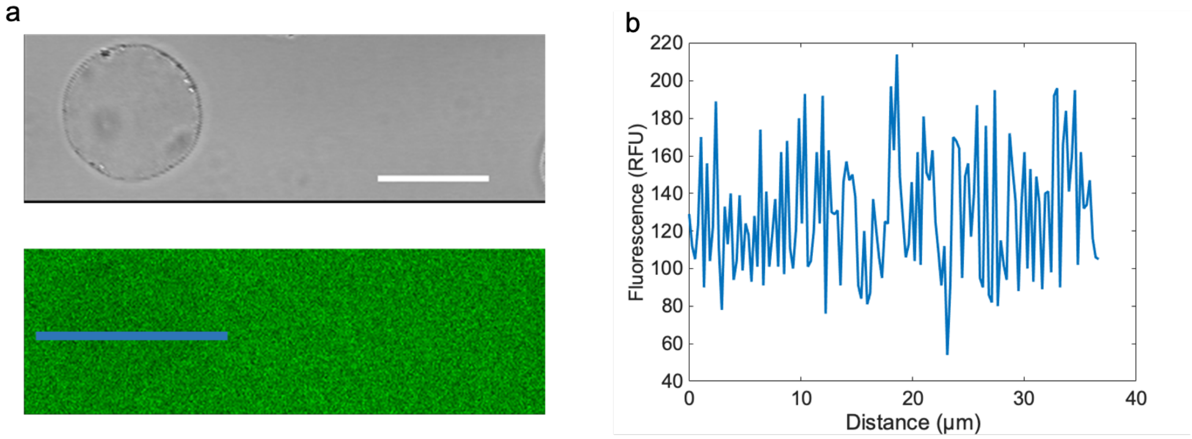

Supplementary Figure 3 : ssDNA is evenly distributed throughout the proteinosome. 10 µM of DY530 labelled ssDNA was added to a dispersion of proteinosomes. (a) Bright field image of a proteinosome (top) and its corresponding fluorescence image (bottom). Blue line indicates the cross-section plotted in (b) using FIJI. Line profile shows an even distribution of DNA within the proteinosome and in the outer aqueous phase. Images are a representations from two replicate experiments. Scale bar is 20 µm. Source data are provided as a Source data file.

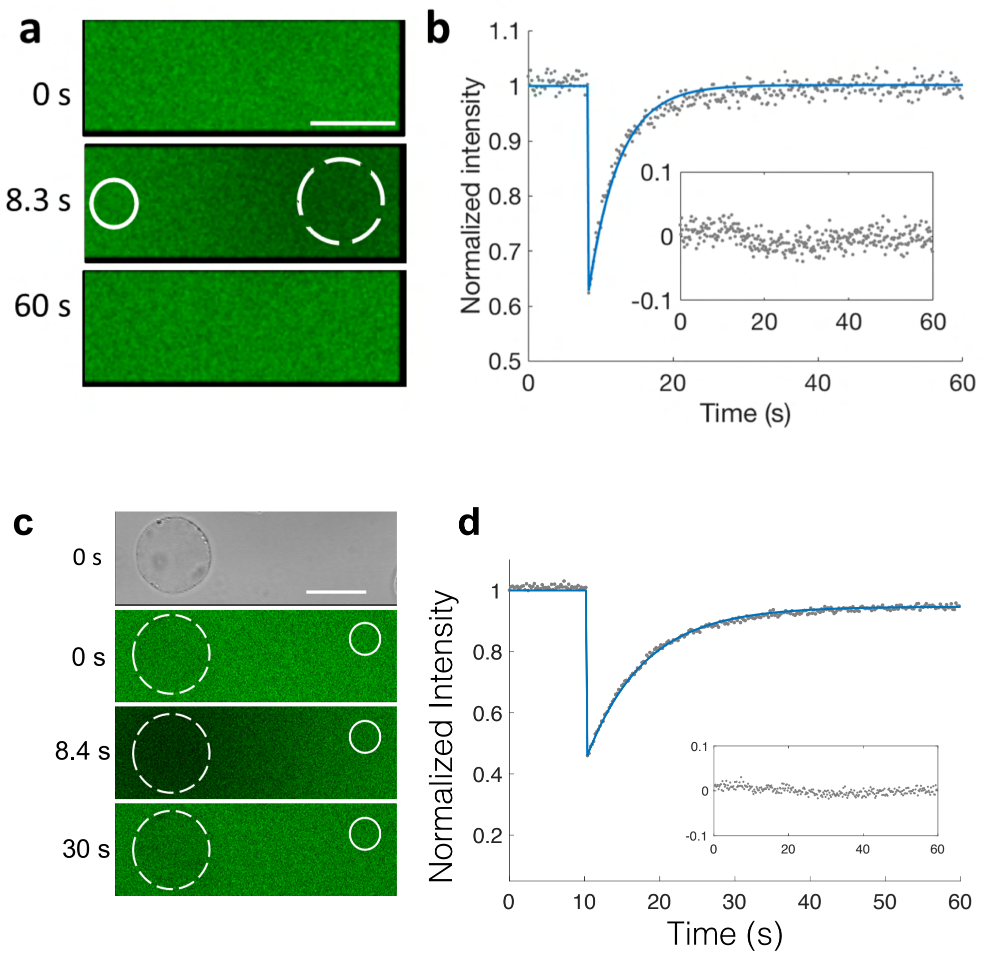

Supplementary Figure 4. FRAP experiments for the determination of diffusion coefficient of ssDNA labelled with DY530. (a) Confocal time-lapse images of fluorescence recovery after photobleaching (FRAP) experiment of a solution containing 10 µM 20-mer ssDNA labelled with DY530. The dotted circle indicates the photobleached spot and the non-dotted circle is the reference. Images are representations from repeat experiments. White scale bar is 20 µm. (b) FRAP data obtained from six experiments. The recovery profile was fit to standard equations and the calculated diffusion coefficient was averaged from the three experiments to give the mean and standard deviation 7.1 ± 1.0 $\mu m^2.s^{-1}$. (c) Confocal time-lapse images of fluorescence recovery after photobleaching (FRAP) experiment of dispersions of proteinosomes incubated with 10 µM of DY530 labelled DNA. The dotted circle indicates the photobleached spot and the non-dotted circle is the background. Scale bar is 20 µm. (d) FRAP data obtained from two experiments. The recovery profile was fit to standard equations and the calculated mean diffusion coefficient was 8.0 ± 1.4 $\mu m^2.s^{-1}$. The error was obtained from the standard deviation. Source data are provided as a Source data file.

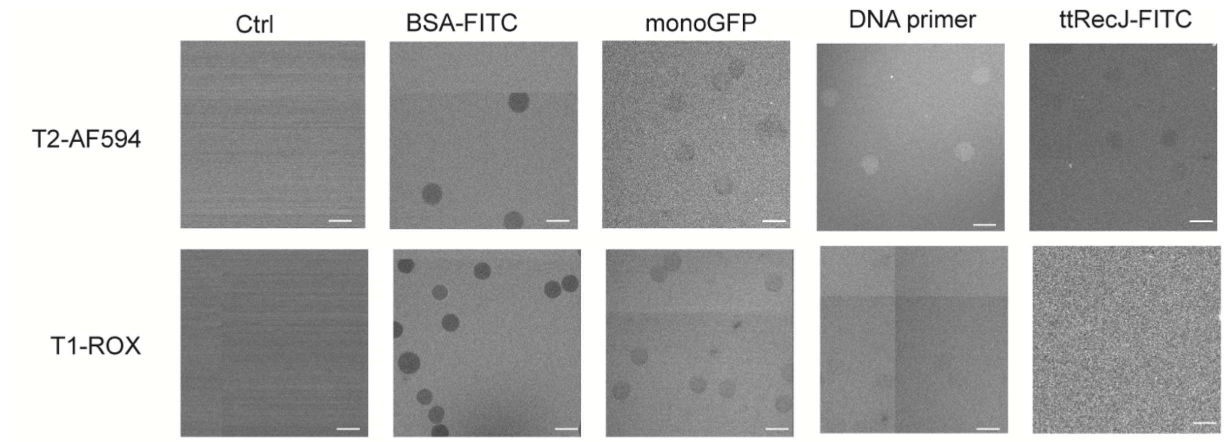

Supplementary figure 5. Partitioning assay of proteinosome with fluorescently labelled molecules. The experiment was performed by incubating ROX-T_1 or AF594-T_2 (dye labelled DNA templates) encapsulated proteinosomes with fluorescently-labelled molecules (alcohol dehydrogenase (ADH), BSA, Formate dehydrogenase (FDH), monoGFP, DNA oligos, ttRecJ) in the reaction buffers at 42˚C. Confocal microscopy images showing the FITC/GFP/FAM channel for the molecular partitioning for the control experiment, no fluorescently labelled molecules, BSA-FITC (0.3 mg/ml (AF594-T_2), 0.51 mg/ml (ROX-T_1), mono-GFP (0.5 mg/ml), FAM-TCGAGTCTGTT (2.5 μM) and ttRecJ-FITC (0.09 mg/ml). Scale bar is 30 μm. The number average molecular weight (M_n) of PNIPAAm used to prepare these proteinosomes was 15000 g/mol, the molecular weight (M_W) 17000 g/mol and its dispersity index ($Đ = M_W/M_n$) was 1.13. The ratio of protein: PNIPAAm was 1: 5.7. The partition coefficient was determined for at least 30 different proteinosomes from two different experiments and the average and standard deviation (error) obtained.

		**ROX-T_1-Biotin**			**AF594-T_2-Biotin**	
**Sample name**	Mw (kDa)	Concentration	Partitioning Coefficient		Concentration	Partition coefficient
**ADH-FITC**	141	0.06 mg/ml	1.09 ± 0.10		0.06 mg/ml	1.01 ± 0.17
**BSA-FITC**	67	2.55 mg/ml	0.97 ± 0.12		0.3 mg/ml	0.78 ± 0.17
**BSA-FITC**	67	0.51 mg/ml	0.84 ± 0.04		0.06 mg/ml	0.76 ± 0.16
**BSA-FITC**	67	0.17 mg/ml	0.71 ± 0.05		0.02 mg/ml	0.80 ± 0.10
**FDH-FITC**	41*	0.2 mg/ml	1.08 ± 0.07		0.2 mg/ml	1.08 ± 0.07
**monoGFP**	25	0.5 mg/ml	0.97 ± 0.05		0.5 mg/ml	0.99 ± 0.17
**monoGFP**	25	0.17 mg/ml	0.97 ± 0.10		0.17 mg/ml	0.96 ± 0.07
**FAM-CATTCTGACGAG**	4	2.5 μM	0.98 ± 0.06		2.5 μM	0.93 ± 0.06
**FAM-TCGAGTCTGTT**	4	2.5 μM	0.96 ± 0.11		2.5 μM	1.04 ± 0.10
**ttRecJ-FITC**	73	0.09 mg/ml	0.98 ± 0.06		0.09 mg/ml	0.87 ± 0.06

*monomer unit

Supplementary table 8: Summary of partition coefficients for a range of different proteins of different molecular weights for proteinosomes containing ROX-T_1-Biotin or AF594-T_2-Biotin obtained by confocal fluoresence microscopy.

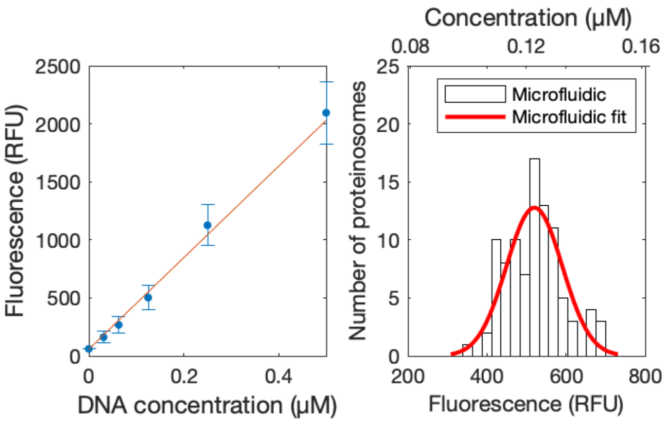

Supplementary Figure 6: Calibration of DNA concentration for proteinosomes produced by microfluidics. (Left) Serial dilution of DY530-labelled DNA-biotin streptavidin under the same microscope settings as the autocatalytic and linear kinetic experiments with proteinosomes. (right) Histogram showing the proteinosomes produced for reactions shown in Figure 3 and 4. Fluorescence units were converted to concentration using the calibration curve and it's linear regression. A gaussian fit to the histogram of number of proteinosomes (n=94) was undertaken to obtain the average concentration 0.12 µM ± 0.02 µM. The total field of view was divided into smaller multiple images and the mean and standard deviation of the fluoresence intensity was obtained from averaging of the fluorescence intensity for the smaller frames across the whole field of view. This was to account for uneven distribution of light across the image. Source data are provided as a Source data file.

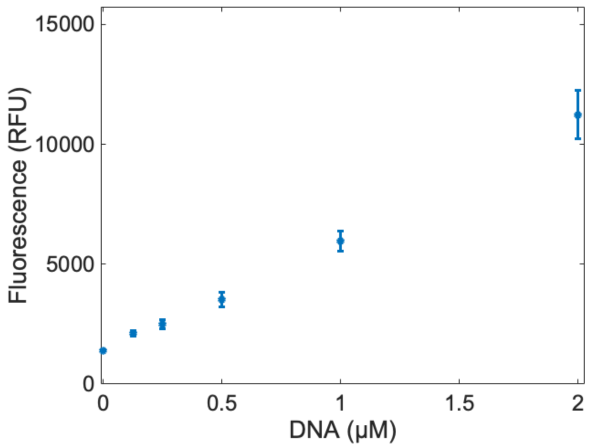

Supplementary Figure 7. Calibration of the fluorescence of 2.2x EvaGreen dye in the presence of 1 µM template with increasing concentration of S_2 DNA. ssDNA solutions were prepared from 0 to 5 µM in the reaction buffer and then incubated at 42°C and their fluorescence measured in a Spark 20 M well plate reader spectrophotometer (Tecan AG, Männedorf, Switzerland) using excitation and emission wavelengths/bandwidth of 470/20 nm and 515/15 nm respectively. Results show increasing fluorescence intensity with increasing DNA concentration. The mean and standard deviation were obtained averaging the fluorescence intensity over 12 minutes. Source data are provided as a Source data file.

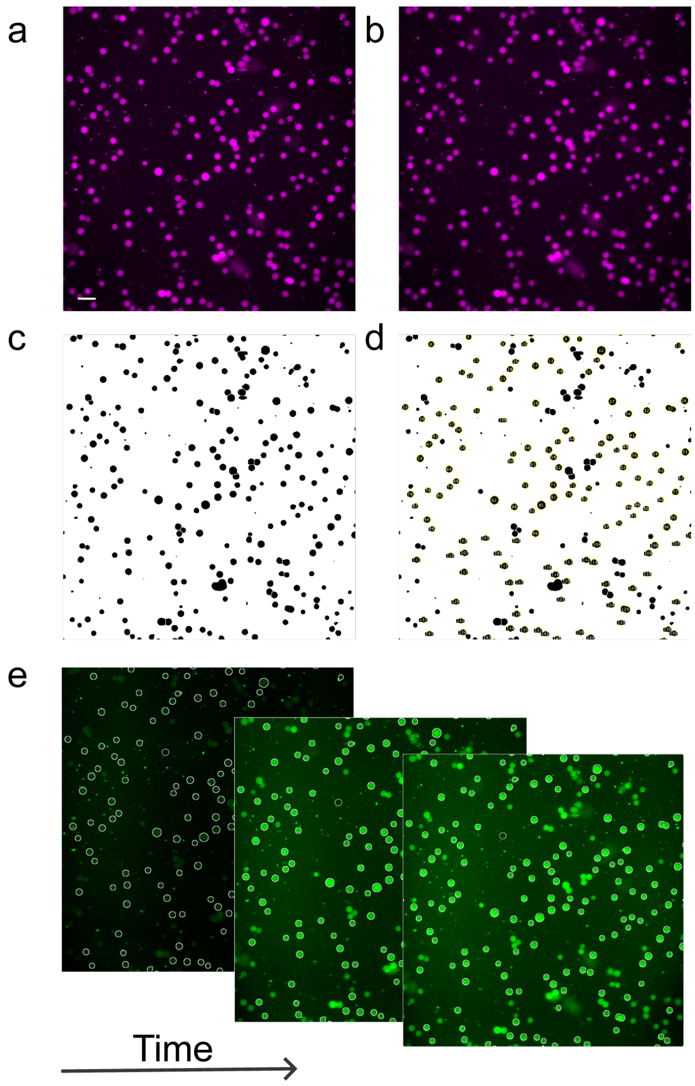

Supplementary Figure 8. Extraction of reaction kinetics from optical microscopy experiments from individual proteinosomes using FIJI. (a) Proteinosomes were located from the raw image from DY530 labelled DNA. (b) The optical image from a was blurred to avoid empty pixels during image thresholding. (c) The blurred image was binarized by thresholding, followed by running the “Watershed” plugin to separate proteinosomes in close contact. (d) The “Analyze Particles” plug-in was used to detect and generate a list of positions in the ROI manager. (e) The mean fluorescence intensity of EvaGreen fluorescence from the individual proteinosomes was obtained for the identified proteinsomes for the time series. The mean fluorescent intensities were used to create kinetic profiles over time for individual proteinosomes. Scale bar 50 μM.

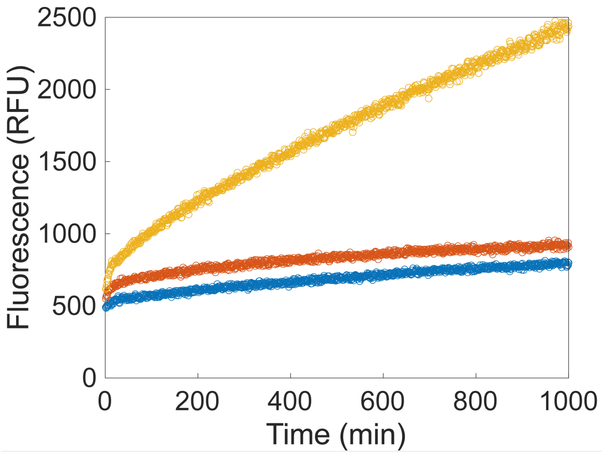

Supplementary Figure 9. Experiment to confirm that all of the template DNA is encapsulated within the proteinosomes. To do this, 7.5 μL of supernatant from a solution of sedimented proteinosomes was mixed with 7.5 μL of buffer solution containing the PEN enzymes and either 0.1 mM dNTPs (blue line) or no DNTPs (orange line) with 2.2x EvaGreen. Alternatively, the proteinosome dispersion was well mixed and 7.5 μl was added to buffer solution containing the PEN enzymes and 0.1 mM dNTPs (yellow line). The fluorescence intensity was measured over time in the well plate reader and results show that there is no DNA template in the surrounding aqueous phase of the proteinosome. This is expected as the proteinosomes are dialyzed using 100 kDa dialysis membrane such that any free-floating 20-mer template is removed from the outside aqueous phase. Source data are provided as a Source data file.

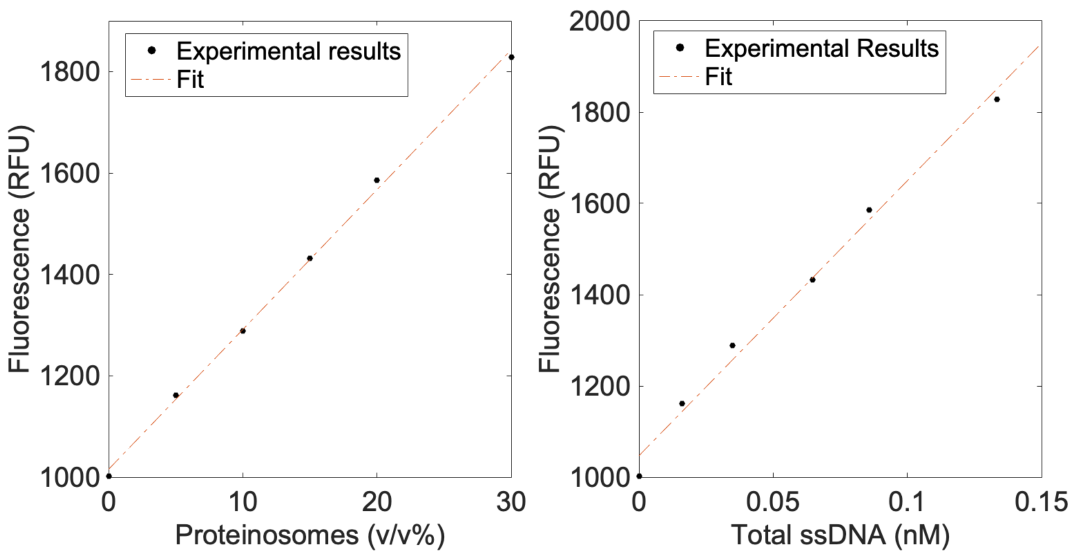

Supplementary figure 10. Calibration curve of the number of proteinosome with encapsulated DNA. The number of proteinosomes (with DNA encapsulated) shows a linear correlation with the fluorescence emitted by the intercalation of 2.2 x EvaGreen with DNA encapsulated within the proteinosomes. Fluorescence readings were carried out at 42°C in a Spark 20 M well plate reader spectrophotometer (Tecan AG, Männedorf, Switzerland) using excitation and emission wavelengths/bandwidths of 470/20 nm and 515/15 nm respectively. The total ssDNA concentration based on the number of proteinosomes ((n= 165 (30 v/v%), n=106 (20 v/v%), n=80 (15 v/v%), n= 43 (10v/v%), n=20 (5 v/v%)) and assuming a 0.12 μM concentration of DNA was encapsulated based on calibration. Source data are provided as a Source data file.

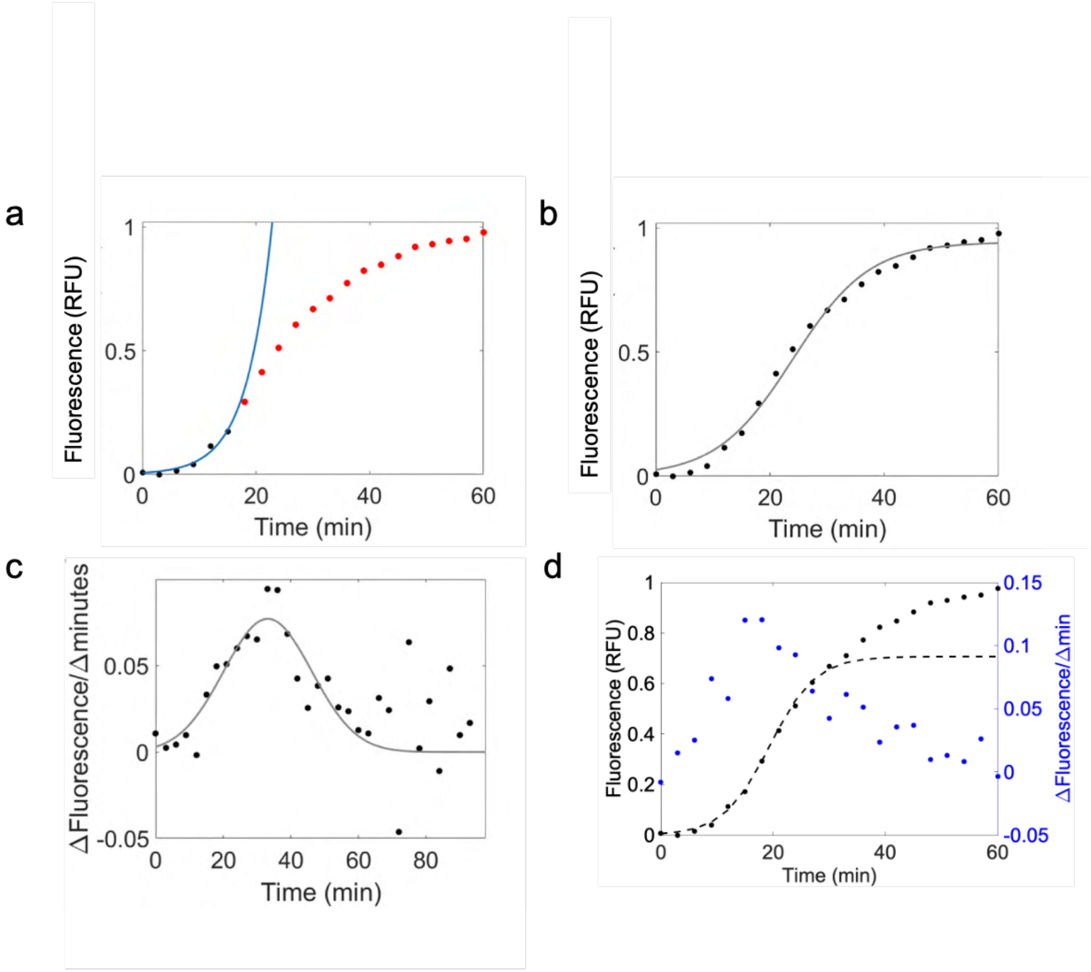

Supplementary Figure 11. An example of a logistic function based fitting methodology to extract exponential reaction rates from autocatalytic reactions. (a) Example of an exponential fitting of autocatalytic data when only a few data points (black markers) can be fitted. This shows that a single extra data point can change the exponential constant significantly. (b) Example of a Logistic model fit through the entire Sigmoidal data set shows that the reaction kinetics at the selected conditions and template sequence are not properly captured by the Logistic model. (c) Discretely differentiated autocatalytic data is fitted to a Gaussian model to find the maximum differentiated rate. (d) The logistic model is fitted from time zero to where the Gaussian function (d) is half the value, after its maximum for all data sets. This results in a good fit with more data points in the area of interest, which is the initial growth, thus providing a simple empiric algorithm with an exponential component.

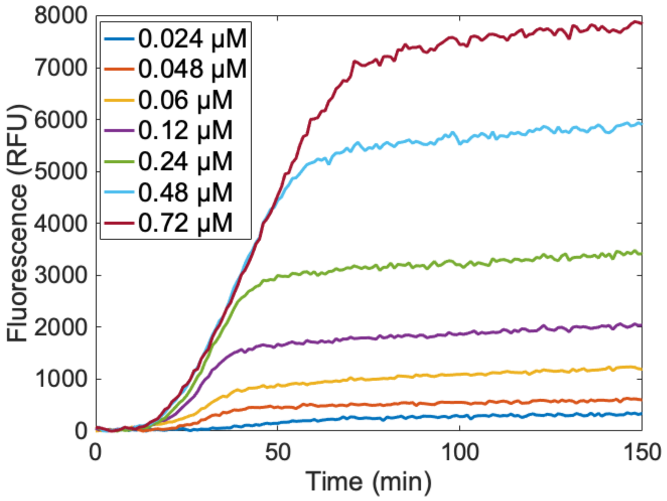

Supplementary figure 12. Reaction rates for autocatalytic reactions carried out with templates in a buffer (b) Autocatalytic kinetic curves show the production of DNA from templates in solution at template concentrations ranging from 0.024 µM to 0.72 µM. The reactions were triggered with 1 nM primer. Fluorescence readings were carried out at 42°C in a Spark 20 M well plate reader spectrophotometer (Tecan AG, Männedorf, Switzerland) using excitation and emission wavelengths/bandwidths of 470/20 nm and 515/15 nm respectively. Source data are provided as a Source data file.

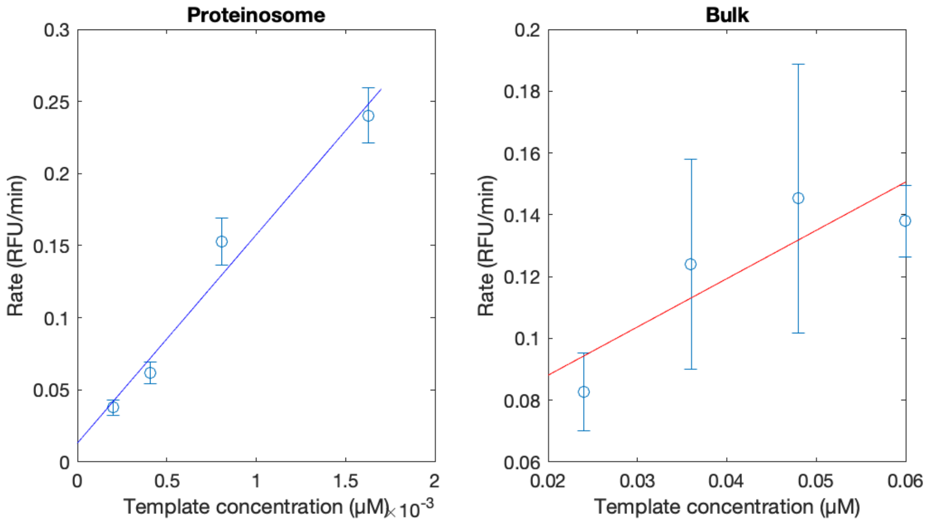

Supplementary figure 13. Linear fits of reaction rates as a function of template for reactions in proteinosomes (left) and buffer (right). The mean and standard deviation (error bars) arise from averaging multiple proteinsomes (left) and from fitting the kinetic profile (right). Source data are provided as a Source data file.

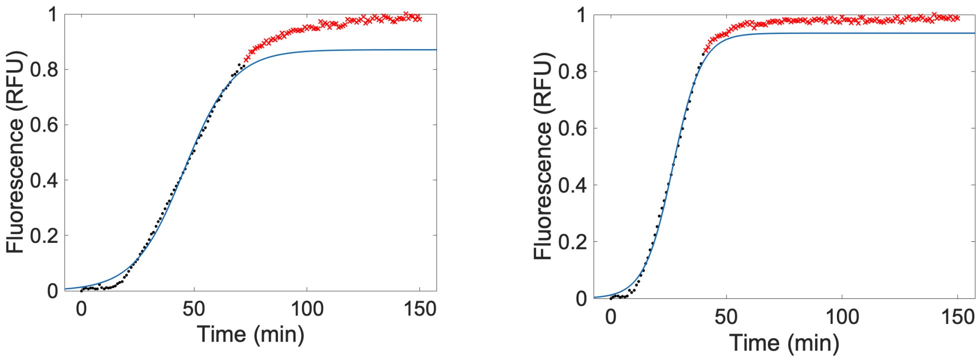

Supplementary figure 14. Determination of the effect of polymerase on the autocatalytic rate of reaction in a buffer solution experiment, with 1 µM template. The autocatalytic reaction was triggered with 1 nM primer and fluorescence readings were carried out at 42°C in a Spark 20 M well plate reader spectrophotometer (Tecan AG, Männedorf, Switzerland) using excitation and emission wavelengths/bandwidth of 470/20 nm and 515/15 nm respectively. (left) Autocatalytic kinetic curves with 12.8 units.mL^{-1} polymerase. Fitting to the data gave an autocatalytic rate of 0.092 ± 0.0004 min^{-1} (right) Autocatalytic kinetic curves with 73.1 units.mL^{-1} polymerase. Fitting to the data gave an autocatalytic rate of 0.16 ± 0.01 min^{-1}. Source data are provided as a Source data file.

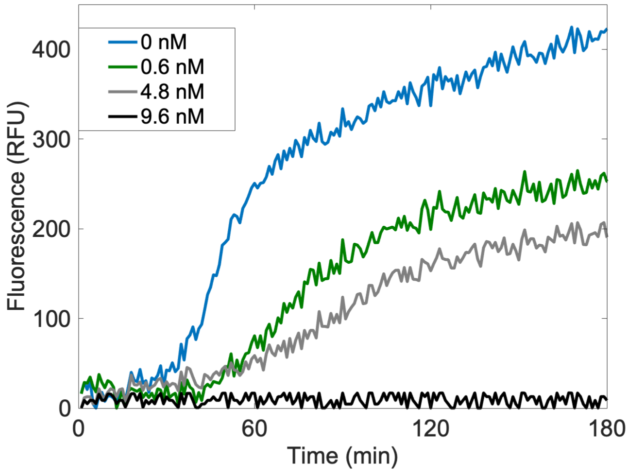

Supplementary figure 15. Autocatalytic kinetics in proteinosomes at different exonuclease concentrations. Proteinosomes containing 0.12 µM template were incubated in the presence of 1 nM DNA primer at 42 °C in a Spark 20 M well plate reader spectrophotometer (Tecan AG, Männedorf, Switzerland) using excitation and emission wavelengths/bandwidths of 470/20 nm and 515/15 nm respectively. Source data are provided as a Source data file.

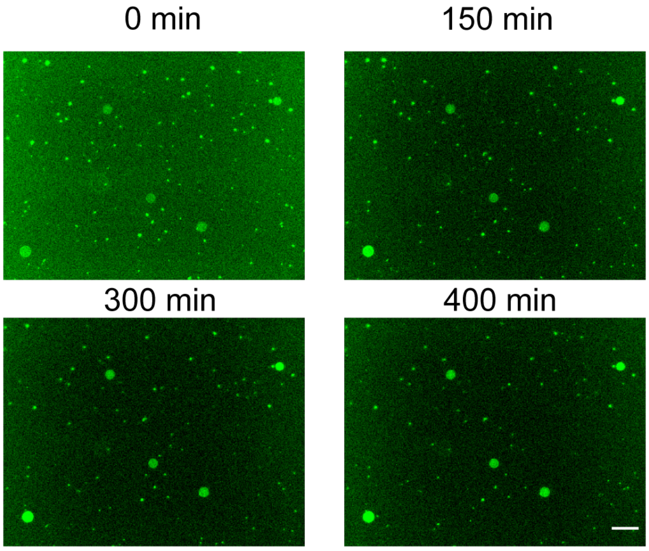

Supplementary Figure 16. Control experiment for the two-node network population. The experiment contained population 2 proteinosomes (final concentration of T_2 was 0.068 nM) and 50 nM of the S_1 primer and no proteinosomes containing T_1. Time-lapse widefield fluorescence microscopy images showing population 2 proteinosomes in the absence of population 1 proteinosomes. Fluorescence comes from the non-sequence-specific intercalation of Evagreen Dye (λ_{ex} = 475/28 nm and λ_{em} = 525/15 nm) into the double-strand. Scale bar is 50 μm. This is a representation from two repeat experiments.

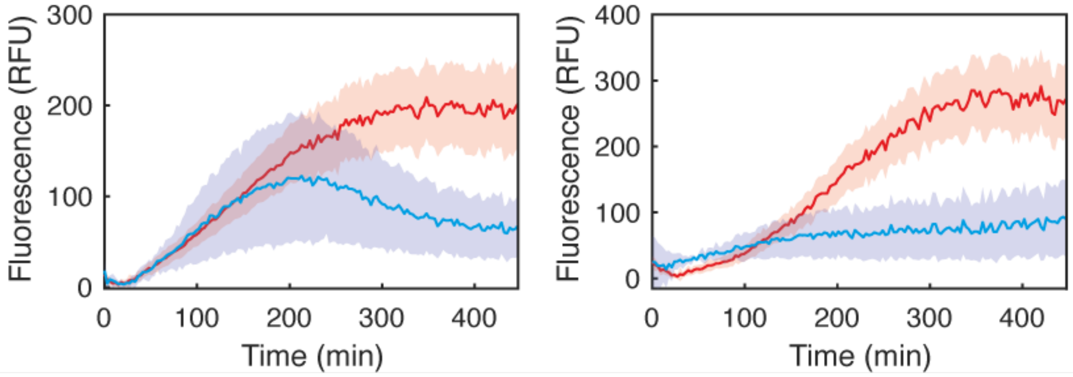

Supplementary Figure 17. Reaction kinetics of a two-node network cell population. Kinetic fluorescence profiles from individual proteinosomes from population 1 (blue curve) and population 2 (red) for a population ratio of 0.5:1 yielding template T_1 and T_2 concentrations of 0.035 nM and 0.068 nM, respectively (left) and 0.25:1 yielding template T_1 and T_2 concentrations of 0.017 nM and 0.068 nM (right). Bold lines represent averaged data from 29 (left-red) and 5 (left-blue) proteinosomes and 23 (right-red) and 5 (right-blue). The shaded colours in the plots are the standard deviations from averaged kinetics. The decrease in the fluorescence intensity from population 1 is due to the action of exonuclease on the primer strand. The location of the two different populations of proteinosomes were identified by two different DNA- labelling dyes (ROX and DY530, ROX- λ_{ex} = 575/15 nm and λ_{em} = 641/75 nm Beamsplitter HC BS 596F and DY 530- λ_{ex} = 542/27 nm and λ_{em} = 593/46 nm Beamsplitter H 560 LPXR. The reaction was measured by detection of EvaGreen (EvaGreen - λ_{ex} = 475/28 nm and λ_{em} = 525/45 nm Beamsplitter H 488 LPXR. Source data are provided as a Source data file.

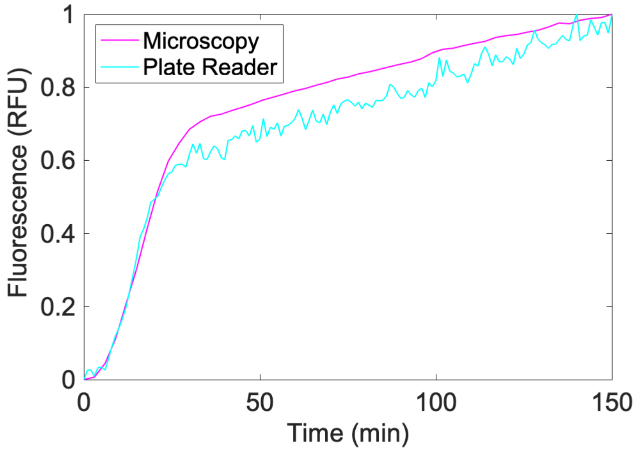

Supplementary figure 18. Normalized autocatalytic kinetics in bulk solution detected in a well-plate reader or in the microscope for the same experiment showing that the normalized data obtained from the plate reader (cyan) and wide-field optical microscope (magenta) correlate to one another. Fluorescence readings were carried out at 42°C in a Spark 20 M well plate reader spectrophotometer (Tecan AG, Männedorf, Switzerland) using excitation and emission wavelengths/bandwidth of 470/20 nm and 515/15 nm respectively. Source data are provided as a Source data file.

Supplementary References:

1. Wakamatsu, T. *et al.* Structure of RecJ exonuclease defines its specificity for single-stranded DNA. *J. Biol. Chem.* **285**, 9762–9769 (2010).
2. Schindelin, J. *et al.* Fiji: an open-source platform for biological-image analysis. *Nat. Methods* **9**, 676–682 (2012).
