# Supplementary material for: Programmable synthetic cell networks regulated by tuneable reaction rates
Source: Nat Commun. 2022 Jul 6;13:3885. doi: 10.1038/s41467-022-31471-5 (PMC9259615; doi:10.1038/s41467-022-31471-5)
Supplement: Supplementary file 2 — Reporting Summary [file 41467_2022_31471_MOESM2_ESM.pdf]

nature portfolio

Corresponding author(s): T-Y Dora Tang

Last updated by author(s): 03/05/2022

Reporting Summary

Nature Portfolio wishes to improve the reproducibility of the work that we publish. This form provides structure for consistency and transparency in reporting. For further information on Nature Portfolio policies, see our Editorial Policies and the Editorial Policy Checklist.

Statistics

For all statistical analyses, confirm that the following items are present in the figure legend, table legend, main text, or Methods section.

n/a	Confirmed	
☐	☒	The exact sample size (*n*) for each experimental group/condition, given as a discrete number and unit of measurement
☐	☒	A statement on whether measurements were taken from distinct samples or whether the same sample was measured repeatedly
☒	☐	The statistical test(s) used AND whether they are one- or two-sided<br>*Only common tests should be described solely by name; describe more complex techniques in the Methods section.*
☒	☐	A description of all covariates tested
☐	☒	A description of any assumptions or corrections, such as tests of normality and adjustment for multiple comparisons
☐	☒	A full description of the statistical parameters including central tendency (e.g. means) or other basic estimates (e.g. regression coefficient) AND variation (e.g. standard deviation) or associated estimates of uncertainty (e.g. confidence intervals)
☒	☐	For null hypothesis testing, the test statistic (e.g. *F*, *t*, *r*) with confidence intervals, effect sizes, degrees of freedom and *P* value noted<br>*Give P values as exact values whenever suitable.*
☒	☐	For Bayesian analysis, information on the choice of priors and Markov chain Monte Carlo settings
☒	☐	For hierarchical and complex designs, identification of the appropriate level for tests and full reporting of outcomes
☒	☐	Estimates of effect sizes (e.g. Cohen's *d*, Pearson's *r*), indicating how they were calculated

Our web collection on statistics for biologists contains articles on many of the points above.

Software and code

Policy information about availability of computer code

Data collection	Custom code will be deposited. The following software was used for data collection: Metamorph microscopy automation and image analysis software (7.10.2.240), Zeiss efficient navigation Zen 2.3 version 14.0.0.21, and 14.0.25.201, 14.0.25.20, Tecan Spark Control Software version 2.3), Microsoft Excel 16.60 (microsoft Office 2019)
Data analysis	Custom code will be deposited on a repository (data statement provided. The following software was used for analysis: Matlab (R2018, R2021B), Microsoft excel version 16.49, Python in jupiterlab version 3.2.1, Microsoft excel 16.60 (Microsoft office 2019)

For manuscripts utilizing custom algorithms or software that are central to the research but not yet described in published literature, software must be made available to editors and reviewers. We strongly encourage code deposition in a community repository (e.g. GitHub). See the Nature Portfolio guidelines for submitting code & software for further information.

Data

Policy information about availability of data

All manuscripts must include a data availability statement. This statement should provide the following information, where applicable:

- Accession codes, unique identifiers, or web links for publicly available datasets
- A description of any restrictions on data availability
- For clinical datasets or third party data, please ensure that the statement adheres to our policy

All data is available upon request and is stored at https://doi.org/10.17617/3.6FGVUS

Field-specific reporting

Please select the one below that is the best fit for your research. If you are not sure, read the appropriate sections before making your selection.

- [x] Life sciences
- [ ] Behavioural & social sciences
- [ ] Ecological, evolutionary & environmental sciences

For a reference copy of the document with all sections, see nature.com/documents/nr-reporting-summary-flat.pdf

Life sciences study design

All studies must disclose on these points even when the disclosure is negative.

Sample size	Sample size was determined by the experiment. For example, the sample size was typically determined by the number of proteinosomes in the field of view and was therefore dependent on the experimental setup. For a given field of view all proteinosomes were taken for analysis. The ROIs are provided in the repository. Sample sizes are given in the figure legends as well as the materials and methods.
Data exclusions	To the best of my knowledge, there were no data exclusions
Replication	Replication was undertaken in a number of ways: experiments were replicated between different experimenters and with different batches of material; experiments were undertaken on different days with different experimental batches. in some cases, technical replicates were undertaken. The number of experiments has been noted in the figure legends. Figure 4c was the result of one experiment due to the difficulty in obtaining exactly the same number of proteinosomes between experiments. Here concentrations were back calculated after analysis of the number of proteinosomes in the sample. Where possible, replications of experiments were undertaken on different days, data was accumulated with overlapping concentrations and allowed us to test the reproducibility of the experiments.<br>Replications of experiments, such as the autocatalysis in proteinosomes alone gave the same general trends that were seen in the experiments that have been reported.
Randomization	NA: Randomization is typically used in patient or biological studies to prevent treatment bias. Which is not relevant in this study.
Blinding	NA:Blinding was not used in these studies because no patient studies were undertaken for this work.

Reporting for specific materials, systems and methods

We require information from authors about some types of materials, experimental systems and methods used in many studies. Here, indicate whether each material, system or method listed is relevant to your study. If you are not sure if a list item applies to your research, read the appropriate section before selecting a response.

Materials & experimental systems

n/a	Involved in the study	
☒	☐	Antibodies
☒	☐	Eukaryotic cell lines
☒	☐	Palaeontology and archaeology
☒	☐	Animals and other organisms
☒	☐	Human research participants
☒	☐	Clinical data
☒	☐	Dual use research of concern

Methods

n/a	Involved in the study	
☒	☐	ChIP-seq
☒	☐	Flow cytometry
☒	☐	MRI-based neuroimaging
